# RADFNet: An infrared and visible image fusion framework based on distributed network

**DOI:** 10.3389/fpls.2022.1056711

**Published:** 2023-01-24

**Authors:** Siling Feng, Can Wu, Cong Lin, Mengxing Huang

**Affiliations:** ^1^ College of Information and Communication Engineering, Hainan University, Haikou, China; ^2^ State Key Laboratory of Marine Resource Utilization in South China Sea, Hainan University, Haikou, China

**Keywords:** distributed fusion, multiscale channel attention, edge attention, image enhancement, intelligent agriculture

## Abstract

**Introduction:**

The fusion of infrared and visible images can improve image quality and eliminate the impact of changes in the agricultural working environment on the information perception of intelligent agricultural systems.

**Methods:**

In this paper, a distributed fusion architecture for infrared and visible image fusion is proposed, termed RADFNet, based on residual CNN (RDCNN), edge attention, and multiscale channel attention. The RDCNN-based network realizes image fusion through three channels. It employs a distributed fusion framework to make the most of the fusion output of the previous step. Two channels utilize residual modules with multiscale channel attention to extract the features from infrared and visible images, which are used for fusion in the other channel. Afterward, the extracted features and the fusion results from the previous step are fed to the fusion channel, which can reduce the loss in the target information from the infrared image and the texture information from the visible image. To improve the feature learning effect of the module and information quality in the fused image, we design two loss functions, namely, pixel strength with texture loss and structure similarity with texture loss.

**Results and discussion:**

Extensive experimental results on public datasets demonstrate that our model has superior performance in improving the fusion quality and has achieved comparable results over the state-of-the-art image fusion algorithms in terms of visual effect and quantitative metrics.

## 1 Introduction

Infrared images and visible images are important sensing information for intelligent agricultural systems. The key to intelligent agricultural systems is to utilize perceptual data for intelligent analysis and decision-making. The infrared imaging technology with anti-interference solid ability uses the radiation energy released by the target so it can penetrate smoke, fog, rain, snow, etc., in the environment. However, the visible light sensor uses light reflectivity to image with much spectral information and high-resolution characteristics. As the application range in intelligent agricultural equipment gradually broadens and the perceived information environment is usually changeable, a single image imaging technology cannot sufficiently perceive the environmental information. It results in the inability of intelligent agricultural equipment to perceive enough information, which leads to the failure of intelligent agricultural systems to work regularly. Therefore, it is of great significance to study the complementary use of infrared and visible image imaging technology to enhance the information perception ability of intelligent agricultural equipment (Aamir et al., 2021).

High-quality enhanced images can be obtained by fusing infrared and visible images to improve the information perception ability in intelligent agricultural equipment and meet various subsequent visual tasks for intelligent agricultural systems. As a branch of information fusion, image fusion has played an essential role in computer vision since it can generate more informative images for high-level vision tasks such as recognition (Basak et al., 2022), detection (Wieczorek et al., 2022), tracking (Bhatti et al., 2022d; Yan and Woźniak, 2022), and surveillance (Chen et al., 2021; Chen et al., 2022b). Significantly, infrared and visible image fusion is a considerable problem and has striking advantages. It is a task that aims to integrate salient features extracted from source images into a single image by appropriate methods (Li et al., 2017). Generally, visible images contain texture information with high spatial resolution and often lose effectiveness under dark or extreme environmental conditions. On the contrary, infrared images can highlight thermal targets in low light or severe weather and contain little texture information because of their low spatial resolution. Infrared and visible image fusion can integrate the complementary virtues from infrared and visible images into synthetic images, which not only conform to human visual perception but also adapt to the application in various vision systems (Bhatti et al., 2022a; Bhatti et al., 2022b).

According to the abstract degree in image information, image fusion is divided into three levels: pixel level, feature level, and decision level (Ma et al., 2019a). In this work, we mainly study pixel-level image fusion methods because they can retain the information from the source image to the maximum extent. In the past decades, scholars have proposed numerous infrared and visible image fusion techniques. These approaches can be broadly classified into two categories: traditional and deep learning-based methods (Ma et al., 2019a). Most traditional infrared and visible image fusion algorithms belonging to pixel-level fusion directly perform mathematical operations on the image pairs after image registration, which have achieved good performance. However, infrared and visible image fusion methods based on deep learning have emerged with tremendous potential and even better performance in recent years.

The traditional methods, in general, cover five approaches: multi-scale transform methods (MST) (Zhu et al., 2018), sparse representation methods (SR) (Cui et al., 2015; Zhang et al., 2018), saliency methods, subspace methods, and other methods (Gangapure et al., 2018). In general, MST-based methods first decompose the source images into multiple scales, and then the multi-scale features are fused using the appropriate fusion rule. Finally, an inverse operation is performed to reconstruct the fused image. The MST based methods usually adopt Laplacian pyramid transform (LP) (Bulanon et al., 2009), wavelet transform (Wavelet) (Mallat, 1989), nonsubsampled contourlet transform (NSCT) (Da Cunha et al., 2006), edge-preserving filter (EPF) (Farbman et al., 2008), curvelet transform (CVT) (Nencini et al., 2007), and multi-resolution singular value decomposition (MSVD) (Naidu, 2011). Sparse representation methods (SR) generally comprise four steps (Ma et al., 2019a): First, a sliding window strategy is adopted to decompose the source image into several overlapping patches. Then a learned over-complete dictionary is used for sparse coding on each patch to obtain the sparse representation coefficients. Thirdly, a reasonable fusion strategy is designed to fuse sparse representation coefficients. Finally, the learned over-complete dictionary produces a marked effect in reconstructing the fused image using the fused coefficients. Among them, the construction of the over-complete dictionary is key in SR (Ma et al., 2019a). The saliency-based methods can highlight regional activity and significance (Meng et al., 2017; Zhang et al., 2017). The subspace-based methods, including the principal component analysis (Bavirisetti et al., 2017), independent component analysis (Mitianoudis et al., 2013), and non-negative matrix factorization (Kong et al., 2014) can remove the redundant information existing in most natural images by converting high dimensional input images into low dimensional spaces or subspaces. Although the existing traditional fusion methods have indicated great performance, these methods require the highly manual design in decomposition and fusion strategies. Their application is subject to unpredictable constraints in some tasks, and their performance deteriorates when the source images are complex due to the degradation of representation (Chen et al., 2022a).

In the past several years, deep learning has been widely applied in infrared and visible image fusion to solve the shortcomings in traditional fusion methods. The application of deep learning-based methods for infrared and visible image fusion mainly reflects in convolutional neural network CNN-based network frameworks, such as convolutional sparse representation (CSR) and generative adversarial network (GAN). The CNN-based fusion frameworks for infrared and visible image fusion are divided into two categories: the depth extraction for image features and the construction for fusion networks. In depth feature extraction, VGG-19 (Ren et al., 2018), ResNet18, ResNet34, ResNet50, ResNet101, and ResNet152 (Szegedy et al., 2017) have been proposed, among which VGG-19 and ResNet152 are commonly applied. The depth of ResNet152 is deeper than that of VGG-19, and deepening network depth improves the depth features in the image. Nevertheless, the more convolution layer parameter maps cause the problems in increasing the number of parameters, the amount of calculation, and the high requirement for computing hardware. The CSR-based methods generally combine PCNN, wavelet transform, and NSCT to construct a fusion network structure, which has been widely used in infrared and visible image fusion. They can effectively represent the salient features in the source images. However, the local modeling approach adopted by image fusion methods based on sparse representation is prone to lead to two major defects: loss of contextualized information and low tolerance of fault matching. The GAN-based fusion algorithms adopt the CNN network structure as the framework with strong feature extraction ability, significantly improve the fusion quality, and use the confrontation between the source image and the generated image to realize the supervision in the source image on the learning parameters. Ma et al. introduced the GAN in the infrared and visible image fusion task for the first time, namely FusionGAN (Ma et al., 2019b), and then more GAN-based fusion frameworks are proposed (Ma et al., 2020; Li et al., 2021b; Ma et al., 2021). Nevertheless, they are limited by the size of the convolution kernel and the depth of the network, ignoring the correlation between the feature map channels.

Although a variety of networks to improve the performance in image fusion have been proposed by many scholars. The CNN-based network frameworks, such as convolutional sparse representation (CSR), generative adversarial network (GAN), and other many network architectures are applied in infrared and visible image fusion. However, the CNN-based fusion frameworks for infrared and visible image fusion are divided into two categories: the depth extraction for image features and the construction of fusion networks. The extraction for depth features requires a deeper network structure, resulting in weak interpretability, extensive computation, and other problems. The construction of the fusion network is also complex and difficult to control. Although many modelsare superficially similar to RADFNet, they have not abandoned these two categories. To get rid of the dilemma in these two kinds of fusion categories, the RADFNet employs a distributed fusion framework to make the most of the fusion output from the previous step. Two channels utilize residual modules with multiscale channel attention to extract the features from infrared and visible images, which are used for fusion in the other channel. Because it adopts distributed fusion, the fusion network does not entirely rely on the extraction in deep features, and the fusion network is simple to construct, showing strong robustness. The RADFNet solves the limitations from most current fusion networks and shows strong adaptability. The main contributions of our work are summarized as follows:

(A) A distributed fusion framework based on residual CNN (RDCNN) for infrared and visible image fusion is proposed in this paper. The distributed fusion framework is distinct from the existing fusion framework in infrared and visible image fusion. It adopts three channels to realize image fusion, wherein two channels are applied to feature extraction and the other channel realizes feature fusion.
(B) To obtain coarse-to-fine features and compensate edge information for fused images, the attention mechanism is discussed. In this way, the fused images retain more prominent information and lose less edge information from source images.
(C) Two loss functions, including the pixel intensity with texture loss and the structural similarity (SSIM) with texture loss, are designed to train the RADFNet. Through experiments, it is found that networks trained by the two loss functions have their own advantages.
(D) Extensive experiments are conducted on public infrared and visible image fusion datasets. Compared with existing state-of-the-art fusion methods, our fusion framework has a promising even better performance in accordance with visual effect and quantitative metrics. In addition, we perform ablation experiments to verify the function in the corresponding module. Last but not least, unregistered source image pairs are fed into the proposed network, emerging the robustness of the proposed framework.

## 2 Materials and methods

### 2.1 Related works

#### 2.1.1 Distributed fusion architecture

**Figure 1:**
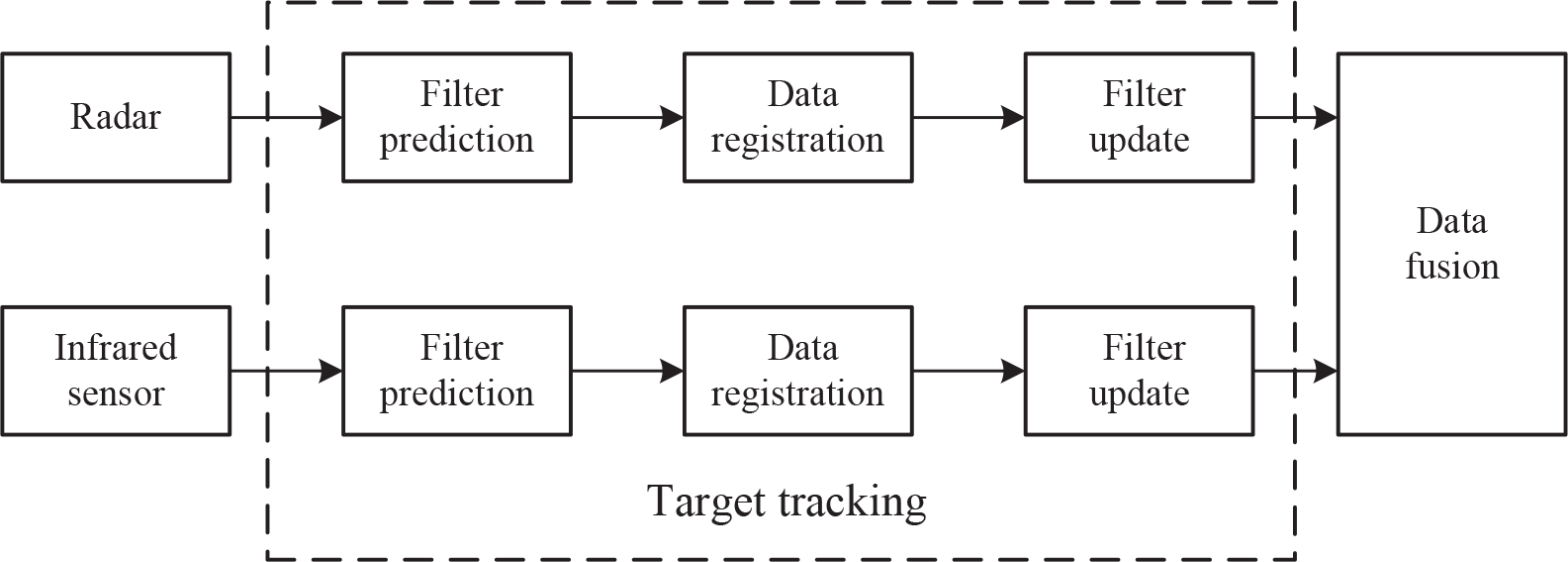
A distributed fusion model for radar and infrared sensors.

Distributed fusion architecture is a classical and typical structure in multi-sensor fusion due to its high speed and reliability (Sun et al., 2017). In distributed fusion, the measurement results of each sensor are processed to obtain local estimates and error covariance. Then the processing results are sent to the fusion node to conflate them into global state estimation and the estimated error covariance (Wu et al., 2021). **Figure 1** shows a distributed model for the fusion in radar and infrared sensors (Yang et al., 2016). For single target tracking, radar and infrared sensors track the target respectively and generate dependent target trajectories in their local information processing center, then send the local trajectory information to the fusion center for data fusion.

#### 2.1.2 Residual network

**Figure 2:**
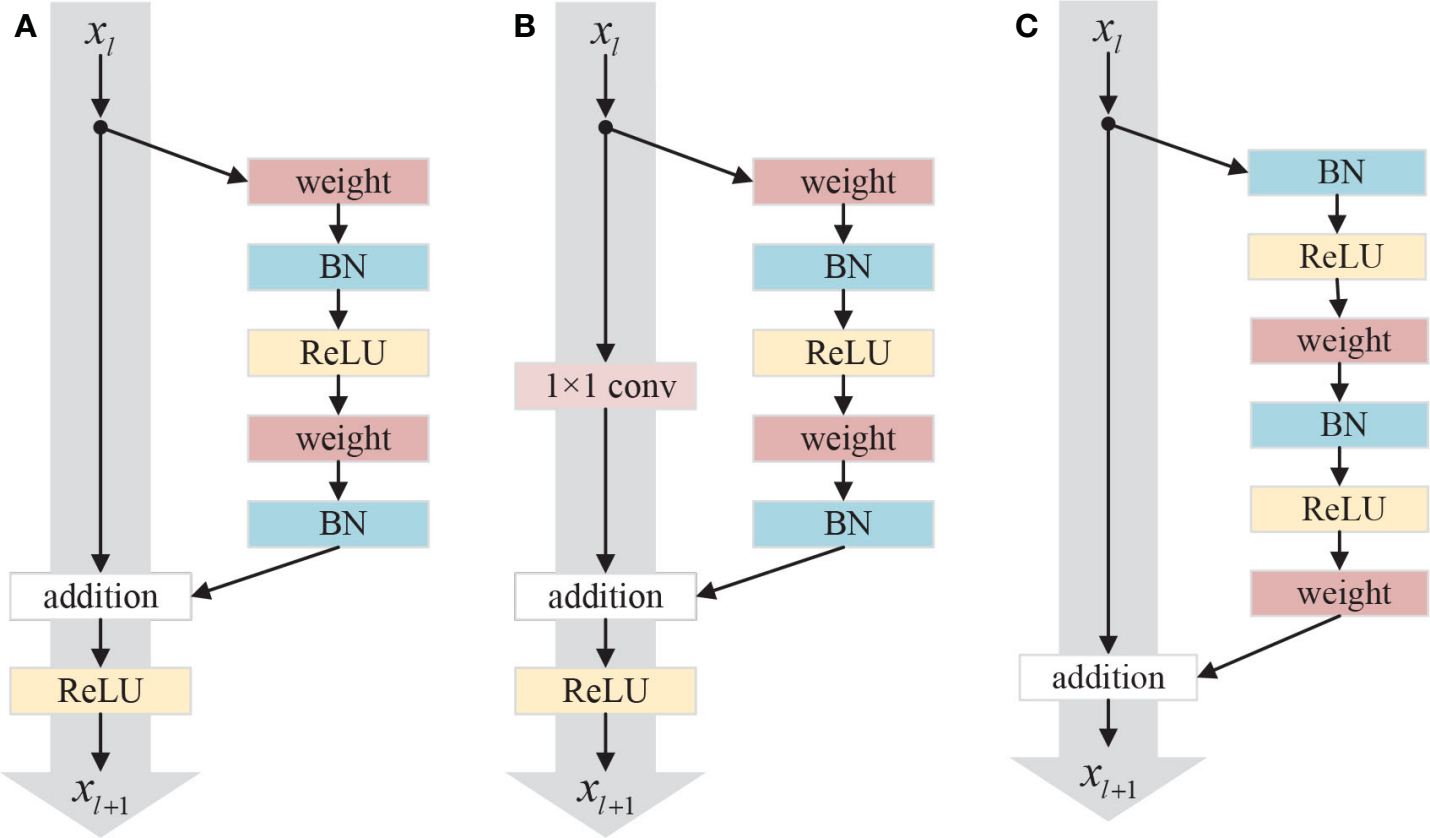
Three different residual units: **(A)** Original residual unit; **(B)** Conv residual unit; **(C)** Improved residual unit.


In some tasks, deeper neural networks can extract higher-level features and perform excellently. However, too deep networks may cause the notorious problem of vanishing or exploding gradients and degrade the accuracy. To solve these problems, He et al. proposed a residual network composed of a series of residual blocks (He et al., 2016a). **Figure 2A** shows the original residual module, which can be expressed as (He et al., 2016b).

(1) $$\begin{array}{c} y_{l}=h\left(x_{l}\right)+ℱ\left(x_{l},W_{l}\right)\\ x_{l+1}=f\left(y_{l}\right) \end{array}$$

where *x_l_* and *x_l+1_* are the input and output in the *l*-th layer, and ℱ is the residual function. *f* is a ReLU (Nair and Hinton, 2010) function. The residual block contains two parts: identity mapping and residual mapping. The left part of **Figure 2A** is the identity mapping, and the right part of **Figure 2A** is the residual part expressed as ℱ(*x_l_*, *W_l_*), which usually contains 2 or 3 convolutional layers. In many cases, the dimensions of input *x_l_* and output *x_l+1_* are discrepant, so it is necessary to employ a 1×1 convolution operation to maintain the dimension in input and output consistent, whose schematic diagram is shown in **Figure 2B**, which can be expressed as (He et al., 2016b).


(2) $$\begin{array}{c} x_{l+1}=h\left(x_{l}\right)+ℱ\left(x_{l},W_{l}\right)\\ h\left(x_{l}\right)=W_{l}^{'}x_{l} \end{array}$$

where h(*x_l_*) is the identity skip connection and $W_{l}^{'}$ is the 1×1 convolution kernel.

The residual network can be formulated as (He et al., 2016b)


(3) $$x_{L}=x_{l}+\sum_{i=l}^{L-1}ℱ\left(x_{i},W_{i}\right)$$

for any deeper block *L* and any shallower block *l*. The formula 3 indicates the feature *x_L_* in any deeper residual block *L* which can be represented as the feature *x_l_* in any shallower block *l* add the residual function, which leads to nice backward propagation properties that the gradient of layers will not vanish even when the weights are arbitrarily small (He et al., 2016b). Moreover, experiments with the various usages of activation function were carried out in (He et al., 2016b). The order of the activation function in the network will affect the performance of the residual network. The structure of the improved residual unit shown in **Figure 2C** has the best performance. In this structure, the batch normalization (BN) and ReLU activation function are placed before the convolution layer, and the activation function after addition is moved to the residual part.

#### 2.1.3 Attention mechanism in deep learning

**Figure 3:**
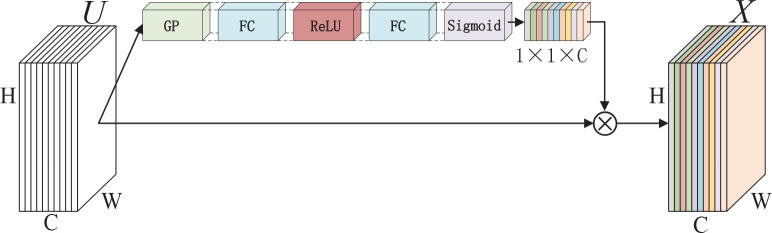
A Squeeze-and-Excitation block, where GP means global average pooling, FC refers to fully-connected layers, ReLU refers to the ReLU function, and Sigmoid refers to the sigmoid function.

Attention mechanism can be traced to the last century, which was mostly applied to machine translation tasks. It has become an essential concept in artificial intelligence because it conforms to some laws of human cognition and can improve the interpretability of neural networks. Therefore, the attention mechanism is widely applied, such as natural language processing, speech recognition and computer vision (Mnih et al., 2014; Vaswani et al., 2017; Bhatti et al., 2022c). In the computer vision domain, many researchers have studied attention mechanism and proposed corresponding methods to acquire nice performance. A residual attention network built by stacking attention modules is proposed in (Wang et al., 2017) which are designed to generate attention-aware features, achieving outstanding recognition performance. A novel architecture unit termed the **“**Squeeze-and-Excitatio**”**(SE) block that adaptively recalibrates the channel feature strength by explicitly modelling the interdependence between channels is introduced in (Hu et al., 2020). The structure of SE block is shown in **Figure 3**, where U is a feature map with the size of W×H×C, ⨂ and refers to channel-wise multiplication, so X and U have the same size. Moreover, edge-guided attention mechanisms which can produce visually appealing images also attract the attention of many researchers (Bhatti et al., 2021). Zhao et al. (Zhao et al., 2019a) propose an edge guidance network (EGNet) which solves the problems of rough boundary in object detection through the complementarity of the object and salient edge information.

### 2.2 Methods

#### 2.2.1 Overall framework

**Figure 4:**
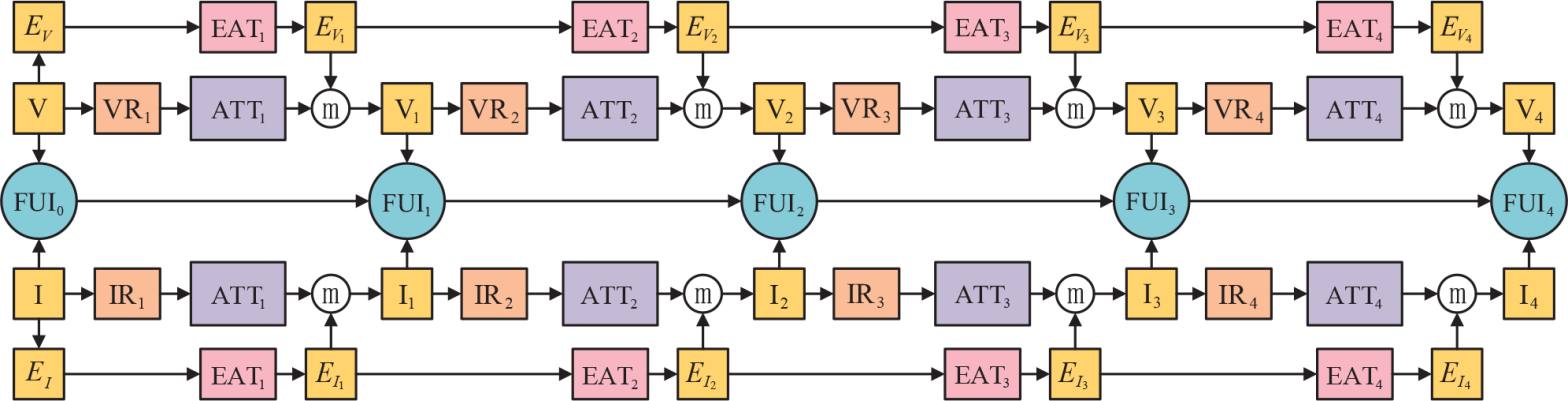
The overall structure for infrared and visible image fusion.

Enlighted by the advantages of distributed structure and the residual module, we propose a novel distributed fusion architecture for infrared and visible images based on the residual module and attention of edge and multiscale channel, RADFNet. The RADFNet is an end-to-end fusion network, the overall structure of which is shown in **Figure 4**. It contains four parts: the feature extraction for the visible image, the feature extraction for the infrared image, the fusion for features, and the compensation for edge information. The infrared and visible image fusion process is formulated as follows.

The visible image features extraction branch can be formulated as


(4) $$V_{i}=ATT_{i}\left(VR_{i}\left(V_{i-1}\right)\right)ⓜE_{V_{i}}\;i=1,2,3,4$$

(5) $$E_{V_{i}}=EAT_{i}\left(E_{V_{i-1}}\right)\;i=1,2,3,4$$

where *V*_0_, the visible image input in the architecture, is the V in the **Figure 4**. *V_i_* is the representation of *V*_0_ after the residual module, multiscale channel attention and compensation of edge information. *VR_i_* means the residual module acting on the *V_i_*_-1_ and *ATT_i_* is the multiscale channel attention module designed to obtain coarse-to-fine features from the outcome of *VR_i_*_-1_. Vi represents the features in different levels of V_0_ with different scales, wherein V_i_ has a higher level than *V_i-_*_1_ *. E_Vi_* is the edge information feature map obtained by *EAT_i_* with input *E*_*V*_*i*−1__ configured to compensate for the edge information of the feature map achieved by residual module and multiscale channel attention module. ⓜ refers to the maximum value in the homologous channel and position in the feature map. The features in the visible image with separate scales are extracted through the above steps. Then, they are fed into the fusion channel to fuse at each layer, which can fully utilize the multi-scale information from perceptible images. In this method, more texture information with high spatial resolution retains, which can enhance the quality of the fused image.

The infrared image feature extraction branch can be formulated as


(6) $$I_{i}=ATT_{i}\left(\underset{i}{\text{IR}}\left(I_{i-1}\right)\right)ⓜ\;E_{I_{i}}\;i=1,2,3,4$$

(7) $$E_{I_{i}}=EAT_{i}\left(E_{I_{i-1}}\right)\;i=1,2,3,4$$

where *I*_0_, the infrared image input in the architecture, is the I in **Figure 4**. *I_i_* is the representation of *I*_0_ after the residual module, multiscale channel attention and compensation of edge information. *IR_i_* means the residual module acting on the *I_i-1_* and *ATT_i_* is the multiscale channel attention module designed to obtain coarse-to-fine features from the outcome of *IR_i_*_-1_ *. I_i_* represents the features in different levels of *I*_0_ with different scales, wherein *I*_i_ has ahigher level than *I_i_*_-1_. *E*_*I*_*i*__ is the edge information feature map obtained by *EAT_i_* with input *E*_*I*_*i*−1__ configured to compensate for the edge information of the feature map achieved by residual module and multiscale channel attention module. ⓜ refers to the maximum value in the homologous channel and position in the feature map. The features in the infrared image with distinct scales are extracted through the above steps. Then they are constituted into the fusion channel to fuse at each layer, which can fully utilize the multi-scale information from infrared images. As a result, rich target information is used for highlighting the target in the fused image.

The channel of feature fusion can be defined as


(8) $$FUI_{i}=\{\begin{array}{cc} F_{i}\left(V_{i},I_{i}\right) & i=0\\ F_{i}\left(V_{i},I_{i},FUI_{i-1}\right) & i=1,2,3,4 \end{array}$$

where *V*_0_ and *I*_0_, which are visible image and infrared image inputs in the fusion architecture, are the V and I in **Figure 4** respectively. *FUI*_1_ *, FUI*_2_ *, FUI*_3_, and *FUI*_4_ are the fusion results with different level features using corresponding rules. *F_i_* refers to the fusion rule of the relevant layer features. *FUI_i_* is the fusion result of the i-th extracted features *V_i_*, *I*_i_ and the different scales from previous fusion result *FUI_i_*_-1_. It realizes the layer-by-layer fusion so it can make the best use of the information from multisource images and then improve the quality of the fused image.

#### 2.2.2 Network structure

**Figure 5:**
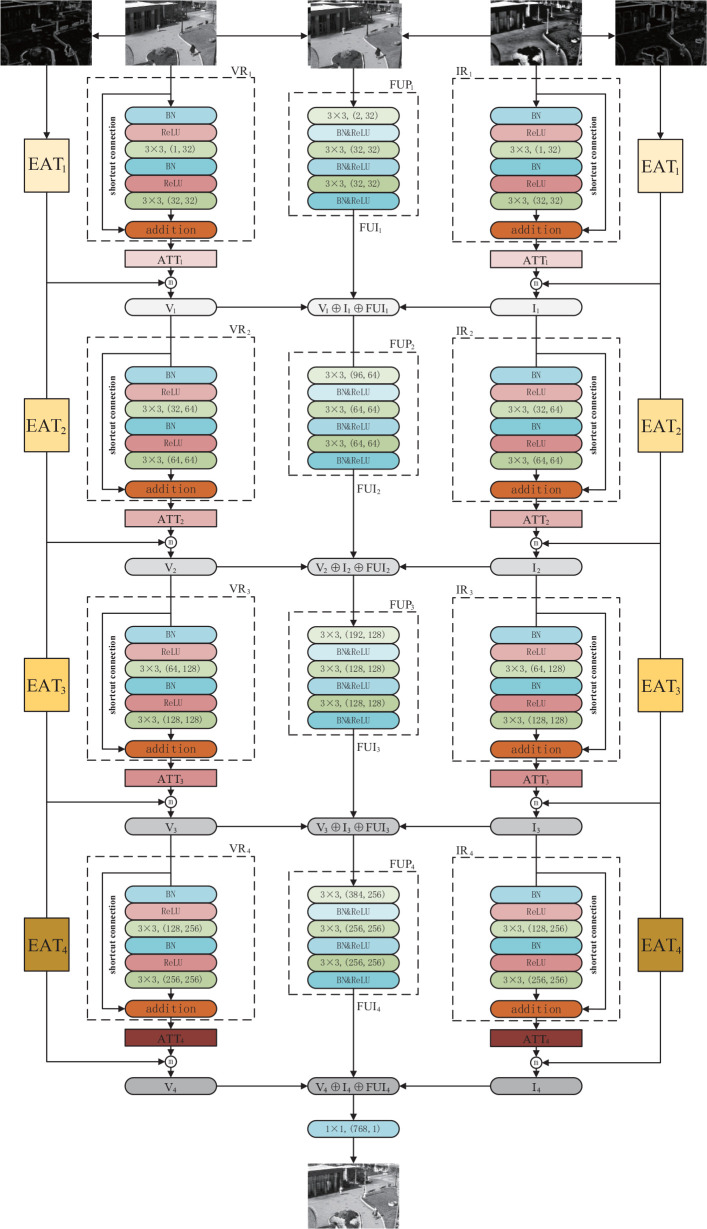
The structure of RADFNet. ‘ATT_1−4_’ denote the multiscale channel attention module and ‘EAT_1−4_’ denote the edge attention module. ‘3 × 3,(1, 32)’ means the kernel size is 3 × 3, input channel is 1 and output channel is 32 in a convolutional layer.

The infrared and visible image fusion model RADFNet set out in the present paper is constituted of three channels. The RADFNet structure is exhibited in **Figure 5**. RADFNet contains four parts: the features extraction branch of the visible image and infrared image, the features fusion branch, and the edge attention module compensating edge information for the extracted features. The left and right branches in **Figure 5** are intended to extract the features in visible and infrared images respectively. The middle branch fuses the features extracted by the two branches with the results from the previous step layer by layer, and the last layer generates the fused image. For a convolutional layer, *‘k×k,(in,out)’* means the kernel size is *k×k*, the input channel is *in* and the output channel is *out*. In the network, BN indicates batch normalization that is utilized to speed up the training and make the training more stable, and ReLU denotes the linear rectification function.

The RADFNet adopts four-layers network structure. The VR_1−4_ and IR_1−4_ are the residual networks which extract image features. Because the residual network has the advantages of mitigating gradient disappearance or gradient explosion and protecting the information integrity, the networks we designed can extract meaningful features and ensure the information integrity simultaneously. Besides, ATT*i* processes the features extracted by residual block VR*i* or IR*i* to obtain coarse-to-fine features. EAT*i* acquires the edge information and then compensates edge information for the extracted feature map. The ⓜ refers to the operation for achieving the maximum value in the homologous channel and the homologous position in the feature map. The FUP*i* generates FUI*i* by fusing features extracted by the other two branches with the FUI_i−1_ generated by FUP_i−1_ when *i* is not 1. When *i* is 1, the concatenated infrared and visible image is fed into the FUP1 to generate FUI_1_. The ⊕ is the concatenation operation in channel-wise, and the 1×1 244 convolution layer in the last fusion layer constructs fusion images.

#### 2.2.3 Multiscale channel attention network

**Figure 6:**
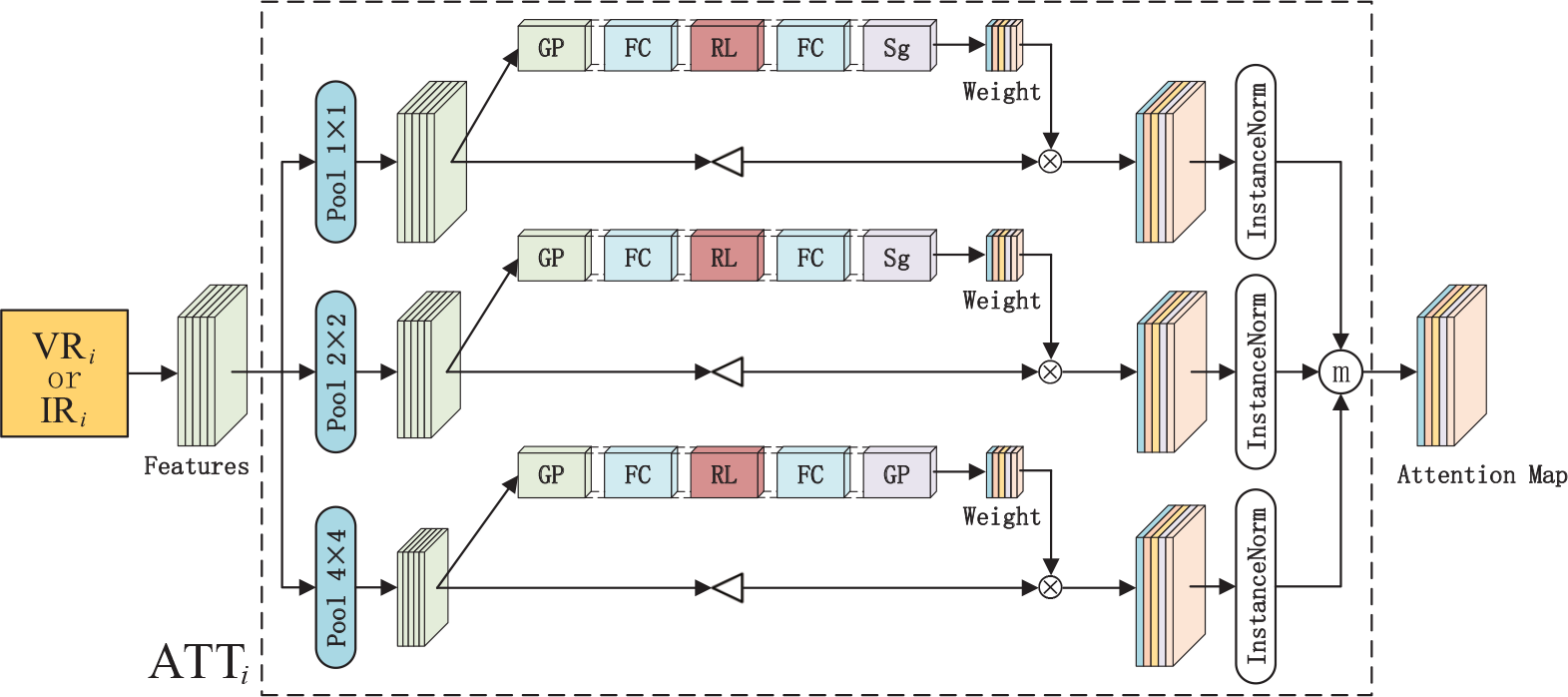
The multiscale channel attention network. The features extracted by residual network(VR*i* or IR*i*) are fed into the ATT*i* to generate attention map. GP, FC, RL, SG denote the global average pooling operation, fully connected layer, ReLUfunction and sigmoid function respectively. ⊲ stands for the up-sample operation and ⨂ denotes the element-wise multiplication.


In the process of infrared and visible image fusion, image feature extraction is exceptionally significant. However, in practical situations, numerous detailed information loses in the process of feature extraction. Inspired by SENet (Hu et al., 2020), the multiscale channel attention network is proposed to process the features extracted by the residual network to obtain the coarse-to-fine features, which can retain more detailed information in the feature map. As shown in **Figure 6**, the structure enclosed by the dotted line is the multiscale channel attention module. The features which lose a lot of details extracted by VR*i* or IR*i* are used as input in ATT*i*. Then, the 1×1, 2×2, and 4×4 average pooling operations are performed to generate multiscale features which contain more necessary spatial information. Moreover, the channel attention mechanism is utilized to enhance channel correlation information between features. The multiscale channel attention network is trained to learn the weight $W_{t_{i}}^{k}$ for the k-th feature $f_{t_{i}}^{k}$ of the t-th pooling scale in the ATT*i* which can be formulated as

(9) $$W_{t_{i}}^{k}=\sigma \left(w_{2}\delta \left(w_{1}G\left(z\right)\right)\right)$$

(10) $$G\left(z\right)=\frac{\sum_{x,y}f_{t_{i}}^{k}\left(x,y\right)}{H\times W}$$

where *G*(*z*) denotes the global average pooling operation. $\sum_{x,y}f_{t_{i}}^{k}\left(x,y\right)$ means the sum of the k-th feature with the *t-*th pooling scale in ATT*i.* (*x*, *y*) refers to the position in feature map, and H,W means the height and width of the feature map. *δ* refers to the ReLU function, *w*_1_∈*ℝ* ^*k*×*k*^ and *w*_2_∈*ℝ* ^*k*×*k*^ , σ denotes the sigmoid function. Then the channel-wise multiplication is implemented between $W_{t_{i}}^{k}$ and the up-sampled features which can be expressed as $UP\left(f_{t_{i}}^{k}\right)$ , ensuring the multiscale features have the same size as the input. Based on this, the reweighted features are obtained and then the attention map can be achieved as follows:


(11) $$F_{i}=\pi \left(W_{1_{i}}^{k}*UP\left(f_{1_{i}}^{k}\right)\right)ⓜ\;\pi \left(W_{2_{i}}^{k}*UP\left(f_{2_{i}}^{k}\right)\right)ⓜ\;\pi \left(W_{3_{i}}^{k}*UP\left(f_{3_{i}}^{k}\right)\right)$$

where *π* denotes the instance normalization (Ulyanov et al., 2016) and ⓜ refers to the operation for acquiring the maximum value in the homologous channel and position in the feature map. Through the above method, the coarse-to-fine attention map *Fi* is obtained. The attention map not only emphasizes more critical features and neglects secondary ones but also reserves more necessarily detailed information.

#### 2.2.4 Edge attention module

Generally, the edge information of an image refers to the sudden change in local grayscale value, color component and texture structure. The edge information from images which is helpful to distinguish objects, can effectively attract attention of people due to human visual characteristics. Enlightened by previous work, we utilize an edge feature map extraction model from the shallower to deeper to obtain the enhanced edge maps, which are designed to compensate for textural information for the fused image.

For the sake of acquiring the edge information used to compensate fused images, we obtain the gradient map from the source images. The process of obtaining the gradient maps ∇*g* by inputting a gray-scale image *f* with the size *h* **×** *w* is defined as


(12) $$\nabla g=\sum_{x=1,y=1}^{x=h-1,y=w-1}\sqrt{{\left(\nabla g^{h}\left(x,y\right)\right)}^{2}+{\left(\nabla g^{w}\left(x,y\right)\right)}^{2}}$$

(13) $$\begin{array}{c} \nabla g^{h}\left(x,y\right)=f\left(x,y\right)-f\left(x+1,y\right)\\ \nabla g^{w}\left(x,y\right)=f\left(x,y\right)-f\left(x,y+1\right) \end{array}$$

where *f*(*x*, *y*) means the pixel at position (*x*, *y*). Moreover, we perform the enhanced operation to obtain the more obvious gradient information:


(14) $$G=\underset{y\in W}{\max}\;\underset{x\in H}{\max}(\nabla g\left(x+1,y+1\right),\nabla g\left(x,y\right)).$$

where *H*={1,…,*h*–1} and *W*={1,…,*w*–1}. The (*x*, *y*) represent the position at the gradient map. Through the above steps, we get the gradient image G with the abundant enhanced edge information.

**Figure 7:**
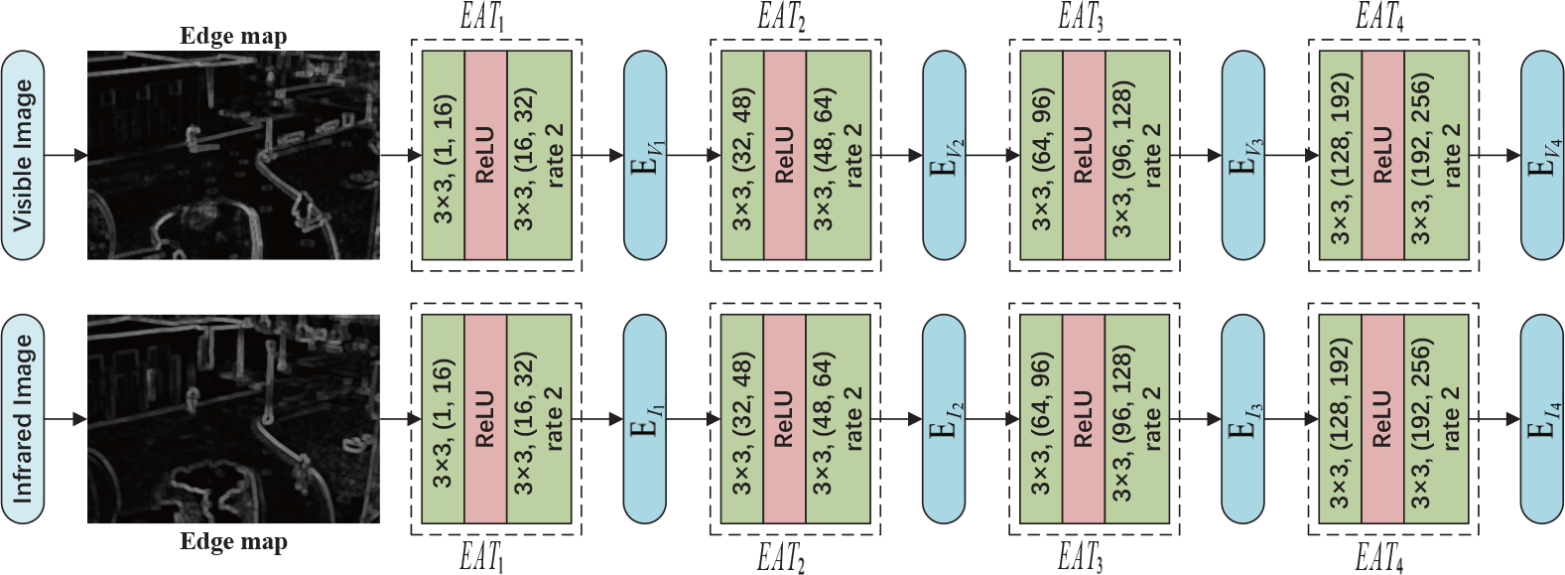
The architecture of edge attention module. The *EAT*_1-4_ are designed to generate shallow to deep edge feature maps *E*_*V*_1−4__ or *E*_*I*_1−4__. For convolution layer, the ‘*k*×*k*,(*in*,*out*)‘ means that the convolution kernel size is *k*×*k* , the input channel is *in* and output channel is *out*. In addition, the ‘rate 2’ denotes the dilated convolution operator with a dilation rate of two.

Subsequently, we feed the gradient images from infrared and visible images into the edge attention module to generate edge attention feature maps with enhanced edge information. Then, the feature maps will be entered into the extraction branch to compensate edge information for the extracted features by IR*i* or VR*i*. The structure diagram of the edge attention module is shown in **Figure 7**. The edge attention module generates E*_Vi_* and E*_Ii_* layer by layer, which is then used to compensate edge information for the feature maps *V_i_* and *I_i_* extracted by VR*_i_* and IR*_i_* respectively. Therefore, compensated feature maps fused to generate the fused images retain more edge information.

#### 2.2.5 Loss function

For infrared and visible image fusion, it is difficult to provide the ground truth of fused images for networks to train a model. However, the requirement to retain salient target information in the infrared image and the texture information in the visible image is determined. Inspired by this requirement, the loss function we employ is as follows:


(15) $$L_{F}=L_{\text{pixel}}+aL_{\text{texture}}$$

where the *L_pixel_* constrains the fused image to contain more target information from the image pair facilitating target tracking and the *L_texture_* forces the fused images to contain more texture details which can effectively improve the identification of objects in images.

Specifically, the exact definition of *L_pixel_* is expressed as follows:


(16) $$L_{\text{pixel}}=\frac{1}{m}\sum_{j=1}^{m}∥I_{f}^{j}-\max\;\left(I_{ir}^{j},I_{vis}^{j}\right){∥}^{2}$$

where *m* is the batch size that is the number of training samples used in each iteration. The I_f_ means the fused image with the input image pair {*I_ir_*, *I_vis_*}, and the max (·) denotes the element-wise maximum selection. Through the maximum selection strategy, the fused images have the prominent target information.

Moreover, we hope the fused images contain significant target information and simultaneously preserve great textural details from source images. However, the *L_pixel_* has very limited constraints on textural details. Therefore, the *L_texture_* is introduced to force the fused image to retain more textural information and the *L_texture_* is defined as:


(17) $$L_{\text{texture}}=\frac{1}{m}\sum_{j=1}^{m}∥\left|\nabla I_{f}^{j}\right|-\max\;\left(\left|\nabla I_{ir}^{j}\right|,\left|\nabla I_{vis}^{j}\right|\right){∥}^{2}$$

where the *m* is the batch size, the *I_f_* means the fused image with the input image pair {*I_ir_*, *I_vis_*}, and the max (·) denotes the element-wise maximum selection. The ∇ indicates the Sobel gradient operator and the |·| means the absolute operation. The element-wise maximum selection strategy can make the fused images obtain the most significant edge textural information.

## 3 Experimental results and analysis

### 3.1 Experimental configurations

To evaluate the proposed fusion algorithm in many aspects, we conduct extensively qualitative and quantitative experiments on the RoadScene (Xu et al., 2020) dataset. We evaluate the performance of our method by making a comparison with six state-of-the-art approaches, including two Nest-based methods, i.e., NestFuse (Li et al., 2020) and RFN-Nest (Li et al., 2021a), and four CNN-based methods: DenseFuse (Li and Wu, 2018), IFCNN (Zhang et al., 2020), U2Fusion (Xu et al., 2022), and SDNet (Zhang and Ma, 2021). The subjective visual perception system is vulnerable to human factors, such as personal emotion and visual environment, and the fused images using different approaches resemble somewhat. Therefore, there are six evaluation statistical metrics which are selected to quantify the evaluation, including mutual information(MI) (Qu et al., 2002), entropy(EN) (Roberts et al., 2008), visual information fidelity(VIF) (Han et al., 2013), stand deviation (SD), spatial frequency(SF) (Eskicioglu and Fisher, 1995) and average gradient(AG) (Zhao et al., 2019b). MI quantifies the amount of information obtained from the source image by the fused image, and EN assesses the amount of information contained in the fused image based on information theory. VIF mainly computes information fidelity in a fused image, which is in line with human visual perception. SD reflects the contrast of an image based on statical concepts, a larger SD value indicates a higher contrast distribution in an image, and the image carries more information. SF reflects the change rate of image gray scale. AG can measure the fused image clarity, which can be considered that the greater AG, the better the image clarity and the better the fused image quality. EN, SF and SD are reference-free metrics. Moreover, a fusion method with larger MI, EN, VIF, SD, SF, and AG represents better performance.

### 3.2 Details of implementation

In the training process of the RADFNet model, we use images from the OSU (Davis and Sharma, 2007) dataset to construct the training dataset. Due to different imaging sensors, the image pairs in the OSU dataset are not strictly registered resulting in black edges in infrared images. Therefore, we crop both infrared and visible images at the same size 280 × 200. Based on the above operations, we can get 8,544 image pairs. It is worth nothing that the visible images in the OSU dataset are color images, but the infrared images are grayscale. To make the number of channels with the input image pair the same, we perform the process that converts the visible images to grayscale images in advance. Moreover, all images are normalized to [0,1] before being fed into the network to accelerate model convergence. The hyper-parameter of the loss is set as *a* = 10. Adam optimizer (Kingma and Ba, 2015) with *β*1 of 0.9, *β*2 of 0.999, epsilon of 10^−8^, weight decay of 0, the initial learning rate of 0.001 is used to optimize our fusion model with the guidance of loss function *L_F_*. All experiments are conducted on the Quadro RTX6000 GPU and 2.90 GHz Intel(R) Xeon(R) Gold 6226R CPU.

The RoadScene dataset contains color visible images, but we employ the input grayscale images to train the proposed network. To get better visuals in the test phase, we adopt the strategy (Prabhakar et al., 2017) to process color images instead of converting the input color images to grayscale images. Precisely, we first convert the color image to the YCbCr color space, then the infrared image and the Y channel of visible image are entered into the RADFNet. Finally, the fusion result is concatenated with Cb andCr channels from visible image along channel-wise and then converted into the RGB color image. The RGB color image is the result of the proposed network.

### 3.3 Results analysis on RoadScene datasets

**Figure 8:**
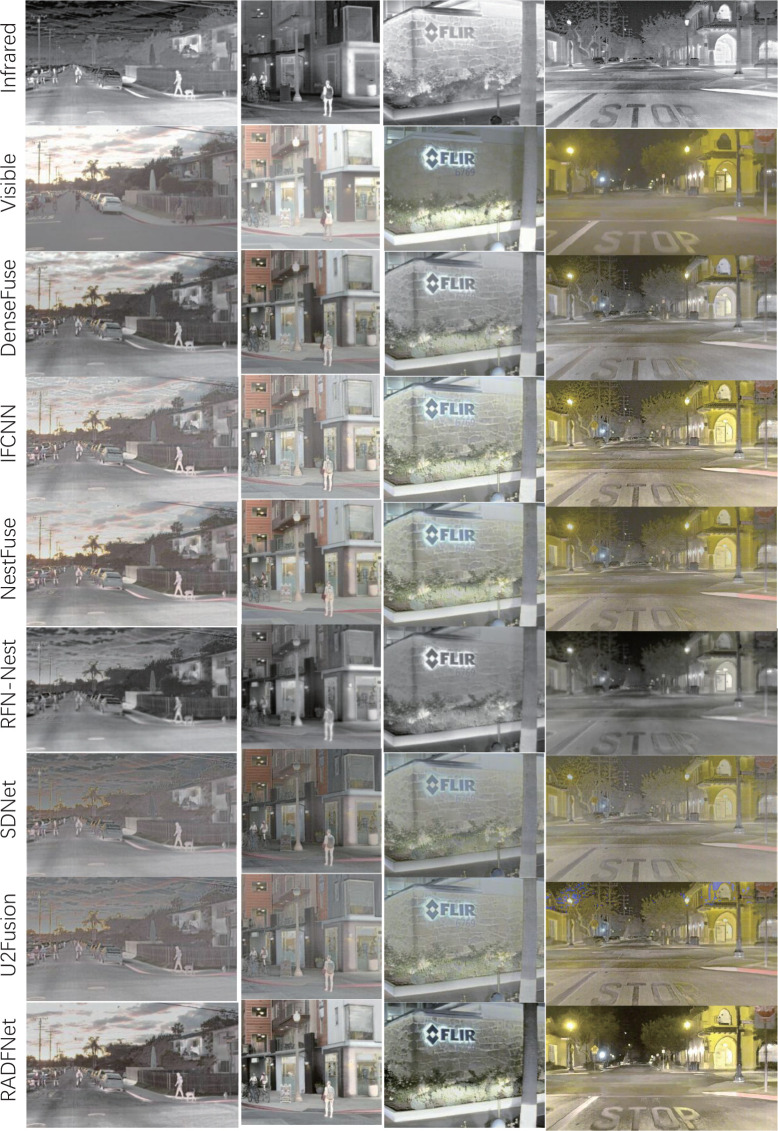
The visual results comparison with different methods on the Roadscene dataset.

To fully evaluate the performance of the RADFNet, we compare the RADFNet with the other six methods on the Roadscene dataset. The Roadscene dataset mainly contains road scenes, including pedestrians and cars, in the daytime and at night. We select two images in the daytime and two in the nighttime for evaluation subjectively so as to exhibit some intuitive fused images on the fusion performance. The fused images of the proposed RADFNet and the other six methods are presented in **Figure 8**. In the daytime scenes, the fused images with exceptional visual quality have rich texture information from visible images and enhanced prominent target information from infrared images. In the first column images in **Figure 8**, RADFNet makes the pedestrians in the image have the most incredible vigorous light intensity. The fused images of U2fusion and SDnet show they tend to darken the entire color of the images. For example, the color of the sky is darker than the fusion images with other methods. In the second column, all six methods enhance the pedestrian. Still, all other methods, except the RADFNet, dim the streetlamp to a certain extent, thus losing information. Moreover, the fusion image of the proposed approach has more obvious color contrast and texture details, so the buildings in our fused image have a richer structure sense than the fused images with other methods. In the nighttime scenes, the ability of both infrared images and visible images to provide information is limited. Therefore, sufficiently retaining meaningful data from the source images is challenging. In the third column, all fusion methods inevitably integrate useless information into the fused image, which degrades the visual quality of the image. Regardless, the proposed approach best protects the information from the visible image while using the meaningful information from the infrared image to enhance the target information. In the last column, compared with other fused images, the fused image in the proposed method failsto remove the halo on the streetlamp altogether. Nonetheless, the signs on the road are most conspicuous in the fusion image, while signs on the street in other images even tend to disappear. In a word, the proposed method can efficiently utilize the information of the infrared and visible images to generate high-quality fused images.

**Figure 9:**
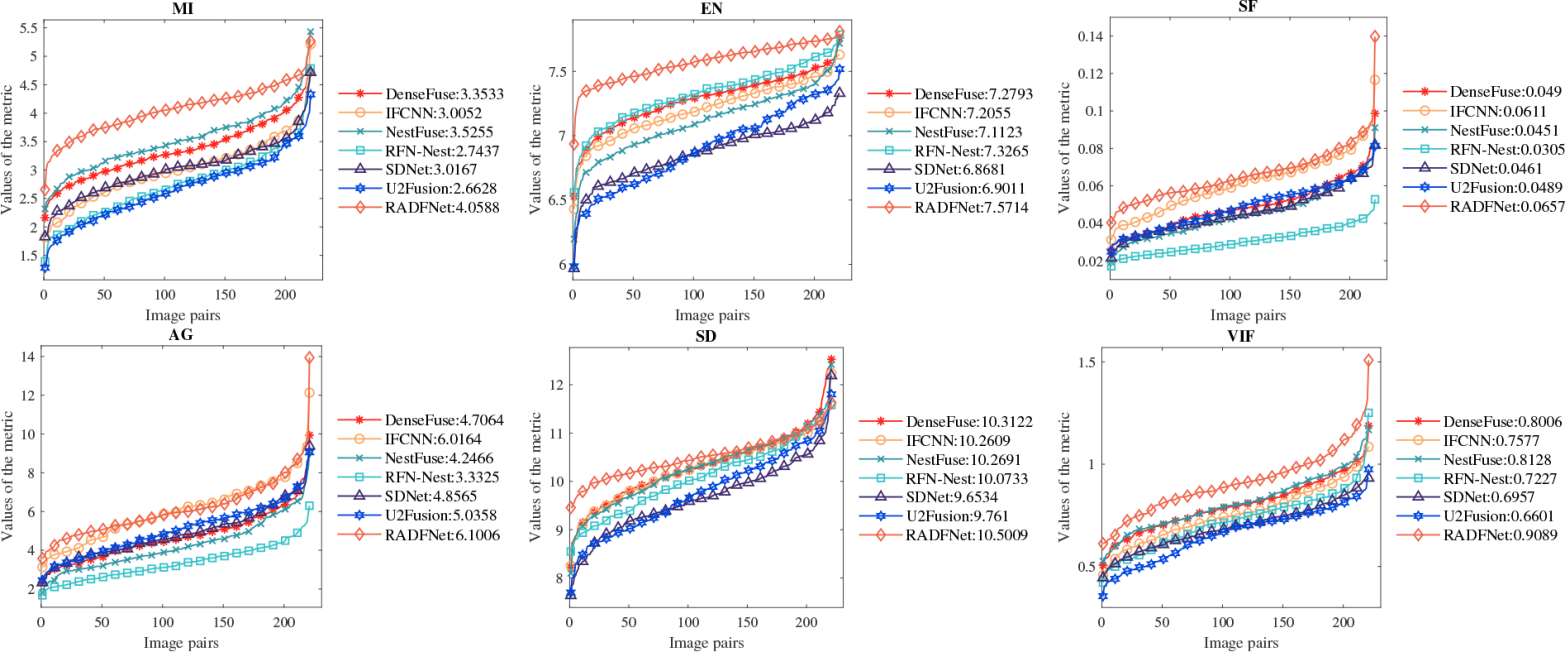
The quantization results of six metrics on the 221 image pairs from the Roadscene dataset. The abscissa x refers to the number of image pairs and the ordinate y refers to the metric value.

To avoid human factors and other factors affecting the subjective evaluation. We conduct quantitative assessments with the six approaches and the proposed method. The results of six metrics on the Roadscene dataset, which contains 221 image pairs, are shown in **Figure 9**. It can be noted that our results achieve better performance on six metrics. The best MI means that our method transfers the most information from the source image to the fused image and the best EN represents the fused image thatcontains the most information. The proposed method represents the best on VIF, which indicates our fused image gets a better human visual perception effect. The best SF and AG suggest that the proposed approach generates the clearest image with remarkable quality. In addition, our RADFNet displays the best SD, illustrating our fused images have the highest contrast. Combined with subjective and quantitative evaluation results, these results prove that RADFNet can convert more meaningful information from infrared and visible images to fused images while ensuring the best quality.

### 3.4 Ablation experiment

**Figure 10:**
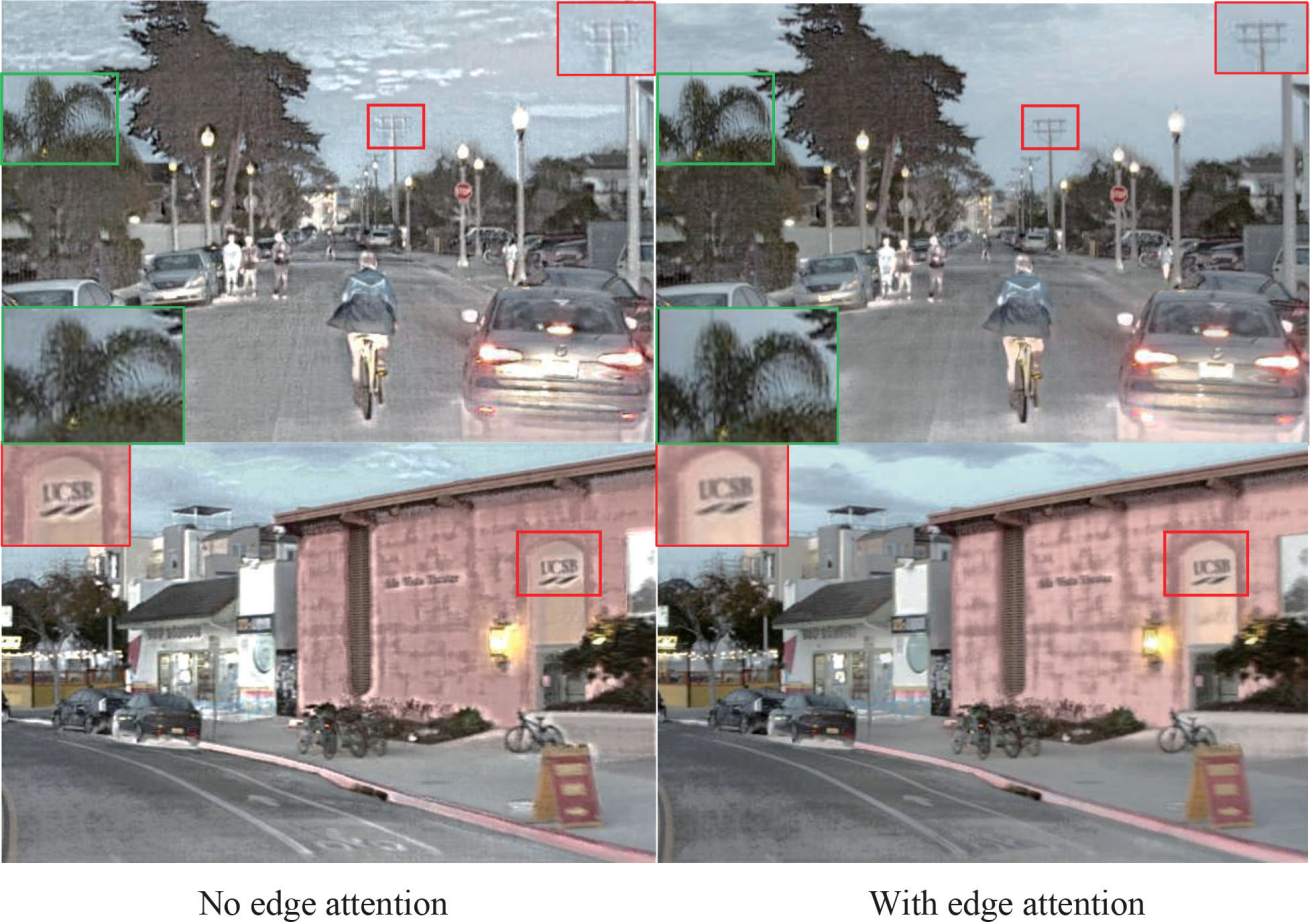
The results of ablation study about the influence of edge attention module in two image pairs from the Roadscene dataset.

To verify the effectiveness of the edge attention module, we conduct ablation experiments. We employ edge attention and ignore edge attention to create two models, then the same image pair is used as input to test the difference between the two models, and the visual results are presented in **Figure 10**. The red and green box parts are magnified for a more intuitive comparison. In the first row, the telegraph pole in the red box with edge attention has a clearer texture, while that without edge attention even becomes blurred. In addition, the leaves with edge attention in the green box also have more precise texture details than that not using edge attention. The words in the red box of the images in the second row are difficult to identify because of the blurred source image. In contrast, words in the fused image using edge attention are more beneficial to observe than that in the image not using edge attention because the edge attention module compensates for the edge information for the fused image.

In addition, to comprehensively evaluate the impact of edge attention in fused images, we make quantitative evaluations for the four images in **Figure 10**, and the result is listed in **Table 1**. It is noted that only the fused images with edge attention have a slightly lower metric SD than that without edge attention. The fused images with edge attention are higher for the other five metrics, i.e., EN, SF, SD, MI, VIF, and AG in both Street and House images. The results show that the generated edge information from the edge attention module compensating for the fused image can improve the image quality effectively.

**Table 1:** The quantitative results on the four images shown in **Figure 10**.

		EN	SF	SD	MI	VIF	AG
Street	Edge	7.514	0.077	10.492	3.890	0.730	7.763
	No-Edge	7.533	0.061	10.721	2.376	0.590	5.905
House	Edge	7.586	0.072	10.334	3.946	0.937	6.846
	No-Edge	7.573	0.056	10.605	2.686	0.709	5.427

The Street means the first row images and House denotes the second row images. Edge and No-Edge refer to edge attention and no edge attention during image fusion, respectively.

### 3.5 Discussion on loss function

For the sake of comprehensively considering the improvement in model training on fused image quality, we design another loss function L_FS_, which can be defined as follows:


(18) $$L_{FS}=\beta L_{SSIM}+L_{texture}$$

where *L_texture_* is represented by Equation 17, the value of *β* is 5, and the *L_SSIM_* is the structural similarity (SSIM) loss, which can be expressed as


(19) $$L_{SSIM}=1-\left(w\cdot \text{SSIM }(F,I\right)+\left(1-w\right)\cdot \text{SSIM }(F,V))$$

where the *SSIM*(·) means the structural similarity (Wang et al., 2004). *F* denotes the output result from the proposed model. *V* and *I* refer to the homologous input visible and infrared images respectively. In addition, to balance the structural similarity loss between the fused image and infrared and visible image, the weight *w* is taken as 0.5.

**Figure 11:**
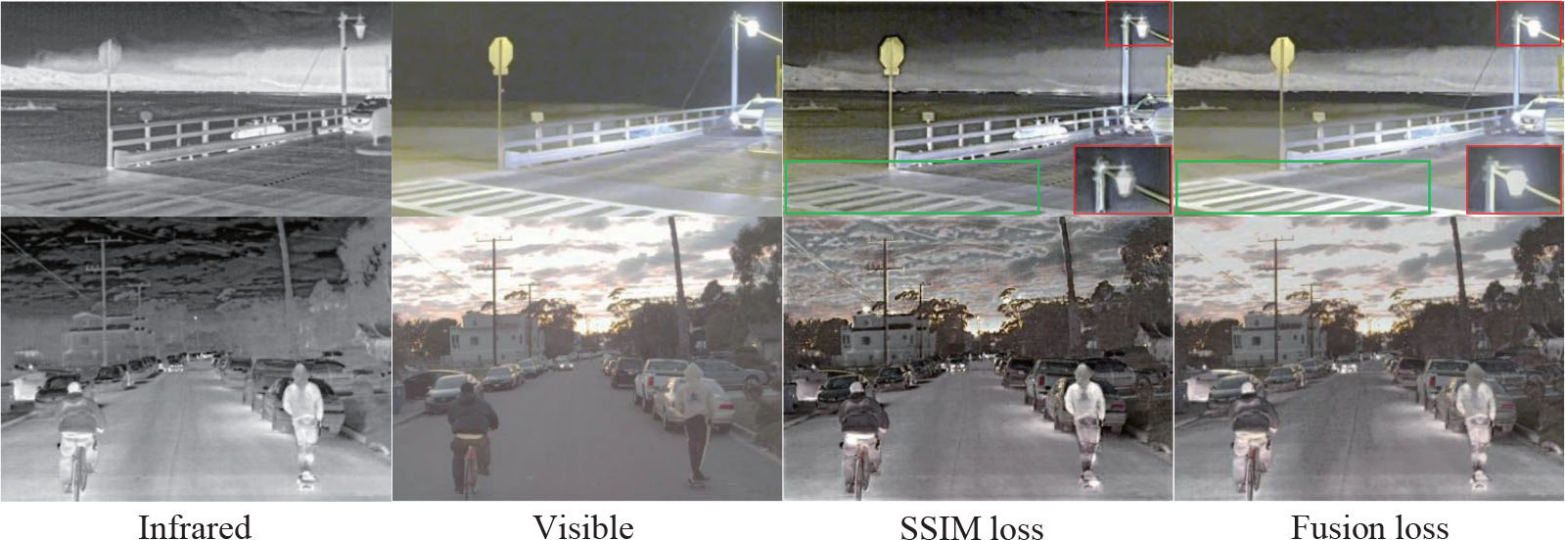
The results of RADFNet trained by SSIM loss *L_FS_* and Fusion loss *L_F_*.

The loss functions *L_F_* and *L_FS_* are used to train the proposed network respectively, and the results are exhibited in **Figure 11**. In the first row, the zebra crossing in the green box of fused image output after the network trained with *L_F_* is more prominent than that trained with *L_FS_*. However, the halo on the streetlamp in the red box in the image output by the network trained by *L_F_* is not completely removed. In the second row, it can be seen that no matter the definition of the whole image or the details, the network output image using *L_F_* training is better. In a word, the output image from the network trained by *L_F_* can highlight more important information in the nighttime scenes. But that trained by *L_FS_* can essentially eliminate the halo in the image. In the daytime scenes, the quality of the output images from the network trained by *L_F_* is better in both overall and detail. Therefore, we choose fusion loss *L_F_* as the training loss function in our experimental test.

To set the best optimal coefficients for the proposed method, the parameter *a* is set as 1, 10, 50 and 100. The epoch and batch size are 4 and 4, respectively. One *a* is needed to choose for the image fusion task based on the test images. Six metrics are employed to evaluate the performance of RADFNet with different *a*. The values are shown in **Table 2**. The best values are indicated in red and the second-best values are denoted in blue. It is worth nothing that three of the six metrics are best when *a*=1. However, the metrics MI and VIF are unstable. When *a*=10, the values of all metrics are considerable and stable, which indicates the proposed network can achieve better fusion performance than other values of *a*. So, *a* is set as 10 in experiments.

**Table 2:** The quantitative results on the RoadScene dataset with different *a*.

	*a=*1	*a*=10	*a*=50	*a=*100
EN	7.612254	7.604088	7.5805	7.58722
SF	0.088493	0.075895	0.076245	0.073816
SD	10.41727	10.50972	10.35863	10.38415
MI	2.670785	3.468535	3.501638	3.156152
VIF	0.698988	0.836942	0.832764	0.787895
AG	8.19272	7.033987	6.88297	6.939895

The red word represents the best, and the blue word represents the second best.

### 3.6 Fusion of unregistered image pairs

**Figure 12:**
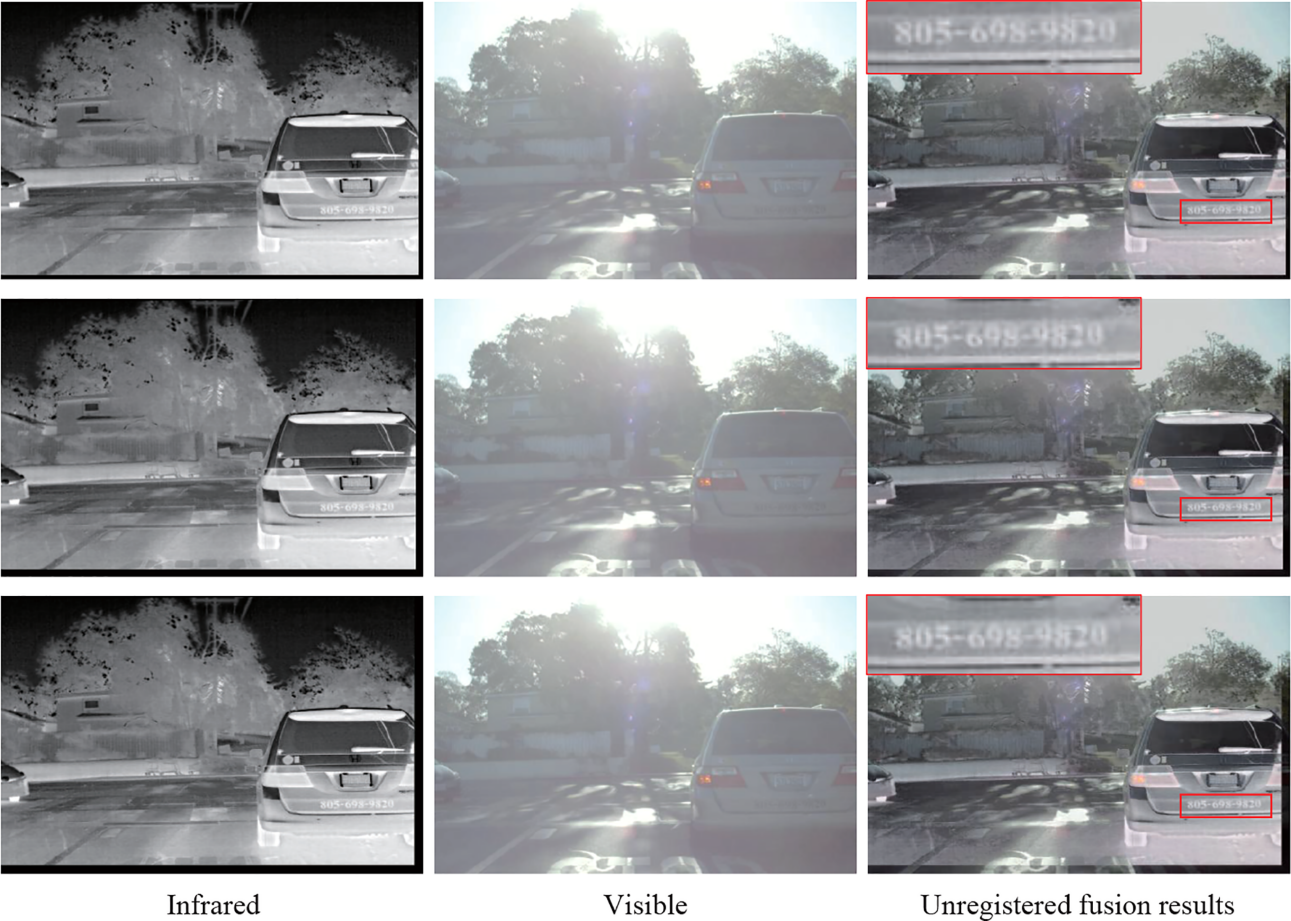
The results of fusing unregistered images with the proposed method on the Roadscene dataset. The infrared images are randomly translated, which causes the black edge in the images. The infrared images from top to bottom are translated with [-5,5],[-8,8],[-10,10] pixels.

In general, it is difficult to obtain the source image pairs that have been strictly registered for image fusion because the imaging characteristics of different sensors are quite different. Therefore, at the training stage, we train our model without using the infrared and visible image pairs that are strictly registered. Aiming to verify that our method performs well in fusing image pairs without strict registration, we randomly translate the infrared images in the source image pairs with [-5,5], [-8,8]and [-10,10] pixels on the Roadscene dataset to get the misregistered infrared and visible image pairs, and then use the proposed method to fuse these misregistered image pairs. The fusion results of these unregistered images are displayed in **Figure 12**. From these fusion results, the proposed method can preserve the target information from the source image. At the same time, the texture details from the source images are also fused into the fused image, which improves the quality of the fused image. The numbers in the red box of the fused images are still vivid, even under different unregistered degrees. The experimental results demonstrate the proposed method with strong robustness still has good performance in fusing images without registration.

## 4 Discussion

For the sake of avoiding the impact of changes in the agricultural working environment on the information perception for the intelligent agricultural system, we utilize infrared and visible image fusion to improve the image quality, so that the fused images can be used normally and even efficiently for various subsequent vision tasks in the intelligent agricultural system. Specifically, we propose a distributed fusion architecture for infrared and visible image fusion, termed RADFNet, which fuses images through three channels based on residual (RDCNN), edge attention, and multiscale channel attention. The proposed method can most retain the salient target information in the infrared image and the textural details information in the visible image. In addition, we introduce the multiscale channel attention module, which can extract coarse-to-fine features to preserve more information from source images to fused images. We also adopt an edge attention module that can compensate edge information for the fusedimage to make the fused image lose less edge information from source images. The comparative experiments are conducted on the Roadscene dataset, and the results demonstrate that the proposed method has superior performance in improving the fusion qualityand has achieved comparable results over the state-of-the-art image fusion algorithms in terms of visual effect and quantitative metrics. Finally, we send the unregistered image pairs into our network, and the results demonstrate that our method with strong robustness still performs well in fusing images without registration. The RADFNet performs well for infrared and visible image fusion due to the robust feature extraction ability of the network. The distributed fusion framework endows it with strong robustness, but the network parameters are still relatively large, which is not simple enough in the actual project deployment. In the future, it is necessary to improve the parameters of the network and the actual deployment of the model.

## Data availability statement

The original contributions presented in the study are included in the article/supplementary material. Further inquiries can be directed to the corresponding authors.

## Author contributions

SF is responsible for the writing and theoretical design of the thesis, CW is responsible for the experimental test, and CL and MH are responsible for the verification of the experimental scheme, the analysis of the results and the project funding. All authors contributed to the article and approved the submitted version.
